# Risk Factors for Progressive Fibrosis and Cirrhosis in Patients With Chronic Hepatitis C in India

**DOI:** 10.7759/cureus.64550

**Published:** 2024-07-15

**Authors:** Amar Deep, Shweta Kumari, Sayan Malakar, Suchit Swaroop, Sumit Rungta

**Affiliations:** 1 Medical Gastroenterology, King George's Medical University, Lucknow, IND; 2 Biochemistry, King George's Medical University, Lucknow, IND; 3 Zoology, University of Lucknow, Lucknow, IND

**Keywords:** liver fibrosis, aspartate aminotransferase (ast), alanine aminotransferase (alt), liver cirrhosis (lc), chronic hepatitis c (chc)

## Abstract

Background

Liver cirrhosis (LC) caused by chronic hepatitis C (CHC) infection is a major global public health concern. This study will look at the risk factors for progressive fibrosis and cirrhosis in patients with persistent hepatitis C virus (HCV) infection.

Methods

In this cohort study, a total of 300 patients were included. We collected comprehensive diagnostic records for the entire study group of 200 people with chronic hepatitis C infection. For the comparison, 100 healthy people were recruited and assessed. FibroScan (Echosens, Paris, France) scores were used to categorize liver fibrosis stages: F0-F1 (no or mild fibrosis, <7 kPa), F2 (moderate fibrosis, 7-8.99 kPa), F3 (significant fibrosis, 9-12.49 kPa), and F4 (cirrhosis, ≥12.5 kPa). Their demographic, biochemical, and serological data were evaluated and compared.

Results

Most patients were males (47% females and 53% males). In the CHC group, the mean age of diagnosis was 37.68±11.57 years, whereas in the chronic hepatitis C-related liver cirrhosis (CHC-LC) group, the mean age was 48.89±12.30 years (p=0.01). Compared to normal individuals, CHC patients had higher body mass index (BMI) (22.37±1.89 versus 21.72±1.95, p=0.01), alanine aminotransferase (ALT) (36.70±7.13 versus 82.78±82.53, p=0.01), and aspartate aminotransferase (AST) (34.96±6.04 versus 80.82±91.77, p=0.01). However, compared to the patients with CHC, the patients with LC have lower platelet (PLT) count (1.51±0.78 versus 1.7±0.41, p=0.01) and higher liver enzymes (AST: 117.7±186.9 versus 80.8±91.7, p=0.01; ALT: 86.71±80.24 versus 82.78±82.53, p=0.01). On regression analysis, higher BMI, older age, low hemoglobin (Hb), and higher bilirubin, ALT, AST, and prothrombin time (PT) were associated with LC.

Conclusion

It is imperative to shift toward prevention and early intervention as the new approach to managing patients with HCV-related cirrhosis. Cirrhosis should be suspected in older patients with CHC who are obese and have low platelet counts with higher liver enzymes.

## Introduction

Hepatitis C virus (HCV) is a hepatotropic ribonucleic acid (RNA) virus from the Flaviviridae family of the genus *Hepacivirus*. It is a major cause of chronic liver disease (CLD), affecting an estimated 71 million individuals globally [1]. HCV has a specific host specificity and tissue tropism, and it is transmitted only through direct blood-to-blood contact between humans [2].

In general, HCV infections are acute; however, their infection causes an immune response that, in most circumstances, cannot prevent chronicity and does not protect against reinfection with homologous and heterologous virus strains in the chimpanzee model [3]. The prevalence of fibrosis is on the rise, owing primarily to viral hepatitis caused by hepatitis C and B viruses [4-6].

In India, cirrhosis of the liver presents a significant public health concern. According to the most recent data from the World Health Organization (WHO) in 2017, liver disease accounted for 259,749 deaths in India, constituting 2.95% of total mortality and contributing to 18.3% of global liver cirrhosis (LC)-related fatalities [5,6]. Scant data are available regarding the evolving trends in the etiology of cirrhosis of the liver over time. Given India's resource-constrained environment, understanding these trends is crucial for formulating effective health policies, particularly for mobilizing resources and allocating funds to plan preventive measures, as many of the causes of cirrhosis are preventable. Regional trends in chronic HCV infection and LC are important to formulate health policies for viral hepatitis too. Recent studies have shown that viral hepatitis-related cirrhosis is associated with a higher incidence of hepatocellular carcinoma (HCC) [7,8]. Compared to autoimmune liver diseases and Budd-Chiari syndrome, HCV-related cirrhosis often gets complicated with HCC [9,10]. Hence, identifying risk factors of HCV-related advanced fibrosis is of paramount importance to halt the progression of the disease and the development of HCC. Therefore, in this study, we aim to evaluate the parameters associated with advanced fibrosis and LC among patients with chronic HCV infection. The findings of the study may help public healthcare specialists to address the global problem with more precision and accuracy.

## Materials and methods

Patients and methods

This is a cohort prospective study conducted between October 2018 and December 2021. The study was conducted in the Department of Medical Gastroenterology of King George's Medical University (KGMU), Lucknow. The Institutional Ethics Committee of King George's Medical University issued approval 537/Ethics/R/Cell17. A total of 300 patients were enrolled subsequentially, out of which 100 were chronic hepatitis C-infected patients (noncirrhotic), while the remaining 100 were liver cirrhosis (LC) patients due to HCV infection. We included 100 healthy individuals. All the patient's clinical, hematologic, demographic, and biochemical data were assessed and recorded at baseline on a predesigned questionnaire.

We included adult patients (>18 years) with chronic hepatitis C infection and liver cirrhosis due to HCV. Patients with diabetes mellitus (DM), alcohol-related liver disease (ArLD), liver cirrhosis due to hepatitis B virus (HBV), autoimmune hepatitis, abdominal tuberculosis, kidney diseases, and hematologic disorders; HCV-infected pregnant or lactating females; and patients who were unable to give written informed consent were excluded. Patients with HCC, prior interferon therapy, human immunodeficiency virus (HIV) and HBV coinfection, and liver transplantation were excluded from the study. The study population was divided into two groups based on liver stiffness stages, that is, the chronic hepatitis C (CHC) group with no sign of fibrosis or cirrhosis (n=100) and the chronic hepatitis C-related liver cirrhosis (CHC-LC) group (n=100). The normal control (NC) group comprised 100 healthy controls who were age- and sex-matched subjects (male/female: 60:40).

All patients were asked about risk factors, premedication, and alcohol abuse. Their medical history was reviewed, and they underwent a physical examination, blood tests, a complete abdominal ultrasound, and a liver fibrosis assessment, which was done by transient elastography (TE).

Diagnosis of hepatitis C virus infection

HCV infection was verified through a two-step process, combining serological assays (third-generation enzyme-linked immunosorbent assay) to confirm anti-HCV antibodies and molecular techniques (reverse transcription-polymerase chain reaction or RT-PCR) for identifying viral particles. In the case of positive anti-HCV antibody test results, further confirmation was sought through the analysis of HCV ribonucleic acid (RNA) using a molecular amplification assay. HCV-RNA viral load was isolated from the patient's serum using the Viral RNA Kit (Qiagen, Hilden, Germany). Subsequently, the core region of the virus was amplified by RT-PCR in a thermocycler, employing specific primers compatible with all HCV genotypes [7].

Definition of acute and chronic hepatitis C

HCV-infected patients were categorized into two patient groups, CHC and CHC-LC, and were defined as if the patients have had a hepatitis C virus infection for less than six months; then, the infection will be defined as an "acute" hepatitis C infection; after six months, it is referred as "chronic" hepatitis C infection [11,12].

Diagnosis of liver fibrosis and cirrhosis

A clinician diagnosed LC based on clinical examination, biochemical tests, abdominal ultrasonography, and transient elastography associated with stigmata of chronic liver disease. The cirrhosis was scored according to the FibroScan (Echosens, Paris, France) scoring system [9] with the cutoff cirrhosis being 7.2 kPa or more for moderate fibrosis and 12.5 kPa or more for cirrhosis. During the study period, all cirrhotic-related problems were treated according to established treatment criteria [13,14].

Liver stiffness evaluation

The technical background and examination procedure have been previously documented in the literature [14-16]. These measurements were conducted on the right lobe of the liver, accessed through intercostal spaces, with patients positioned in the dorsal decubitus posture and their right arm maximally abducted. On the day of the outpatient visit, transient elastography (TE) was done in the supine position using the FibroScan 502 (Echosens, Paris, France) following the normal protocol. Measurements were taken between the ninth and 11th intercostal gaps along the middle or posterior axillary lines. Each patient underwent at least 10 measures, and a success rate (the ratio of valid shots to the total number of shots) of more than 80% was judged acceptable. The values are provided in kilopascals (kPa), ranging from 1.5 to 75 kPa. Any measurements lacking the correct vibration shape or appropriate vibration propagation were automatically discarded by the software [15,16]. In this study, stages F0-F1, with a kPa value of less than 7, are considered no or mild fibrosis. Stage F2, with a kPa value between 7 and 8.99, is categorized as moderate fibrosis. Stage F3, with a kPa value from 9 to 12.49, is identified as significant fibrosis. Stage F4, with a kPa value of 12.5 or higher, is classified as liver cirrhosis. These four phases of liver fibrosis are categorized based on the FibroScan score [14-16].

Statistical analysis

The study's findings were analyzed using the SPSS software version 24 (IBM SPSS Statistics, Armonk, NY). Data for quantitative variables are represented as mean and standard deviation (SD) or median and interquartile range (IQR), whereas data for qualitative factors are presented as numbers and percentages. The Mann-Whitney U test, Student's t-test, and χ² test were used for analyzing normally distributed, non-normally distributed, and categorical data, respectively. Spearman's correlation analysis was used to investigate the correlations between variables. A two-tailed test (p<0.05) indicated statistical significance.

## Results

Demographic characteristics

HCV-infected patients were categorized into two patient groups, CHC and CHC-LC, and were defined as if the patients have had a hepatitis C virus infection for less than six months; then, the infection will be defined as an "acute" hepatitis C infection; after six months, it is referred as "chronic" hepatitis C infection [8]. Of 100 normal controls, 43 (43%) were females, and 57 (57%) were males. Of 100 chronic hepatitis C (CHC) patients, 47 (47%) were females, and 53 (53%) were males, and for patients with chronic hepatitis C-related liver cirrhosis (CHC-LC), 41 (41%) were females, and 59 (59%) were males.

Age-sex distribution in CHC (CHC versus CHC-LC)

The median age of the normal control group in this study was 36.91±11.84 years. Males were more frequently infected, with 47% being female and 53% being male. Their biochemical, demographic, and clinical characteristics are presented in Table 1. In the CHC group, the mean age was 37.68±11.57 years, while in the CHC-LC group, the mean age was 48.89±12.30 years (Table 1).

**Table 1:** Comparison of patients' blood parameter distribution in CHC and CHC-LC compared with normal control (NC) p<0.05 and p<0.01 indicate statistically significant values *Number and percentage in the case of male and female CHC, chronic hepatitis C; CHC-LC, chronic hepatitis C-related liver cirrhosis; LC, liver cirrhosis; LSM, liver stiffness measurement

Parameters	NC (n=100)	CHC (n=100)	CHC-LC (n=100)	P-value: NC versus CHC	P-value: NC versus LC	P-value: LC versus CHC
Female versus male	43 (43%) versus 57 (57%)	47 (47%) versus 53 (53%)	41 (41%) versus 59 (59%)			
Age (years)	36.91±11.84	37.68±11.57	48.89±12.30	0.10	0.01*	0.01*
Body mass index (kg/m^2^)	22.37±1.89	21.72±1.95	21.89±3.18	0.01*	0.15	0.63
Hemoglobin (g/dL)	13.40±1.27	12.52±2.33	11.35±2.58	0.01*	0.01*	0.01*
Total leukocyte count (cells/mm^3^)	9,541.60±1,451.84	7,697.32±2,406.69	7,003.98±3,175.43	0.01*	0.01*	0.07*
Platelets (lac cells/mm^3^)	176,430.00±34,830.00	171,008.00±41,136.07	151,670.00±78,224.37	0.19	0.01*	0.03*
Total bilirubin (mg/dL)	0.79±0.4	0.82±0.51	1.74±2.01	0.55	0.01*	0.01*
Aspartate aminotransferase (IU/L)	34.96±6.04	80.82±91.77	117.78±186.91	0.01*	0.01*	0.08
Alanine aminotransferase (IU/L)	36.70±7.13	82.78±82.53	86.71±80.24	0.01*	0.01*	0.71
Alkaline phosphatase (IU/L)	196.23±39.77	242.65±154.54	249.90±154.32	0.01*	0.01*	0.73
Albumin (g/dL)	4.39±0.46	4.32±0.56	3.53±0.71	0.05	0.01*	0.01*
Prothrombin time (second)	13.95±0.77	14.49±5.86	15.89±3.26	0.36	0.01*	0.02*
LSM value (kPa)	5.31±0.88	5.91±0.76	17.52±10.98	0.01*	0.01*	0.01*

The mean FibroScan score of the CHC group was 5.91±0.76 kPa, and the mean FibroScan score of the CHC-LC group was 17.52±10.98 kPa (Table 1). Patients with different stages of liver cirrhosis in the CHC-LC group were found as follows: no (0%) patients in stages F0-F1, 17 (17%) patients in stage F2, 29 (29%) patients in stage F3, and 54 (54%) patients in stage F4, as presented in Table 2.

**Table 2:** Stages of liver fibrosis as per the FibroScan *Statistically significant p-value

FibroScan stages	Reference range	Measured value
F0-F1	≤7 kPa	0* (0%)
F2	7-8.99 kPa	17 (17%)
F3	9-12.49 kPa	29 (29%)
F4	≥12.5 kPa	54 (54%)

Chronic hepatitis C group versus normal control group

When the CHC group was compared with the normal control (NC) group, statistically significant differences in the age and the value of body mass index (BMI) (22.37±1.89 versus 21.72±1.95, p<0.01), hemoglobin (Hb) (13.40±1.27 versus 12.52±2.33, p<0.01), total leukocyte count (TLC) (9,541.60±1,451.84 vs 7,697.32±2,406.69; p<0.01), aspartate aminotransferase (AST) (34.96±6.04 versus 80.82±91.77, p<0.01), alanine aminotransferase (ALT) (36.70±7.13 versus 82.78±82.53, p<0.01), alkaline phosphatase (ALP) (196.23±39.77 versus 242.65±154.54, p<0.01), and FibroScan (5.31±0.88 versus 5.91±0.76, p<0.01) were observed and given in Table 1. The result reveals that the value of different parameters was higher in the CHC group than in the NC group (Table 1).

Chronic hepatitis C-related liver cirrhosis group versus normal control group

Subsequently, it was observed that there were statistically significant (p<0.01) differences in different study parameters between the CHC-LC group and the normal control (NC) group such as age, TLC, platelet (PLT), AST, ALT, ALP, prothrombin time (PT), and FibroScan as presented in Table 1. The analysis of Table 1 revealed that the mean values of age (48.89±12.30 versus 36.91±11.84, p<0.01), Hb (11.35±2.58 versus 13.40±1.27, p<0.01), serum bilirubin (1.74±2.01 versus 0.79±0.4, p<0.01), AST (117.78±186.98 versus 34.96±6.04, p<0.01), ALT (86.71±80.24 versus 36.70±7.13, p<0.01), ALP (294.90±154.32 versus 196.23±39.77, p<0.01), PT (15.89±3.26 versus 13.95±0.77, p<0.01), and FibroScan (17.52±10.98 versus 5.31±0.88, p<0.01) were higher in the CHC-LC group compared to the NC group with p<0.01.

Chronic hepatitis C group versus chronic hepatitis C-related liver cirrhosis group versus normal control group

Further, the CHC group was compared with the CHC-LC group, and statistically significant differences in the value of Hb (12.52±2.33 versus 11.35±2.58, p<0.01), serum bilirubin (0.82±0.51 versus 1.74±2.01, p<0.01), albumin (4.32±0.56 versus 3.53±0.71, p<0.01), and FibroScan (5.91±0.76 versus 17.52±10.98, p<0.01) were observed between the CHC group and the CHC-LC group with p<0.01 (Table 1). The result reveals that the values of different parameters were higher in the CHC-LC group than in the CHC group (Table 1).

A simple correlation analysis was used to determine the distinctive relationship between liver cirrhosis and factors. Consequently, it was discovered that the correlation between the following variables was positive: sex was positive (0.053), age was negative (-0.005), hemoglobin (g/dL) was negative (-0.060), platelets (10³/UL) were negative (-1.174), total bilirubin was negative (-0.035), AST (IU/L) was positive (0.000), ALT (IU/L) was positive (0.000), ALP (IU/L) was negative (-9.381), albumin (mg/dL) was negative (-0.129), prothrombin time (%) was positive (0.178), and the international normalized ratio (INR) was positive (0.352) (Table 3).

**Table 3:** The characteristic correlation between variables and liver cirrhosis BMI, body mass index; Hb, hemoglobin; TLC, total leukocyte count; PLT, platelet; ALP, alkaline phosphatase; ALT, alanine aminotransferase; AST, aspartate aminotransferase; PT, prothrombin time

Variable	Liver cirrhosis	Standard error
Sex	0.053	0.184
Age (years)	-0.005	0.007
BMI (kg/m^2^)	-0.005	0.026
Hb (g/dL)	-0.060	0.037
TLC (cells/mm^3^)	1.093E-5	0.000
PLT (lac cells/mm^3^)	-1.174E-6	0.000
Serum bilirubin (mg/dL)	-0.035	0.044
AST (IU/L)	0.000	0.001
ALT (IU/L)	0.000	0.001
ALP (IU/L)	-9.381	0.001
Serum albumin (g/dL)	-0.129	0.134
PT (seconds)	0.017	0.030
Dependent variable: FibroScan group		

Linear regression analysis on liver cirrhosis

**Figure 1:**
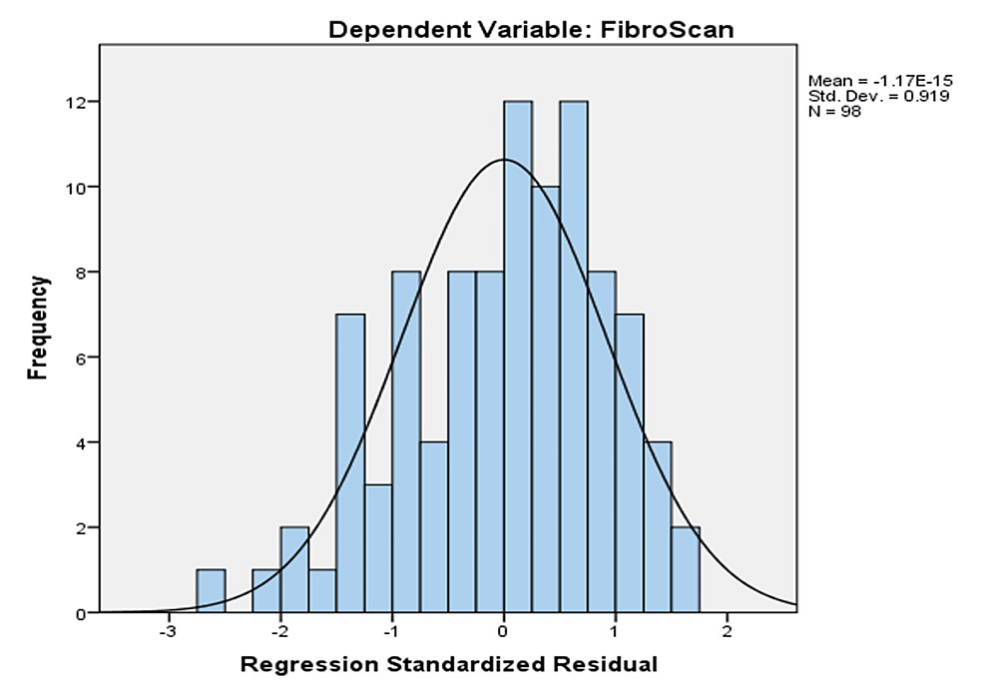
Linear regression analysis on liver cirrhosis

By doing linear regression analysis on liver cirrhosis-related parameters, we were able to calculate the constant, standard error, and significant probability of each variable, as well as the liver FibroScan result (Figure 1).

## Discussion

CHC remains a significant public health challenge in India, particularly in regions such as Uttar Pradesh. Advanced fibrosis and cirrhosis are critical complications associated with CHC, contributing to increased morbidity and mortality. This cross-sectional study identified and evaluated the risk factors for these severe liver conditions in the local patient population.

Liver cirrhosis caused by CHC is a major public health concern that causes significant morbidity and mortality [17]. The severity and course of liver fibrosis determine the prognosis and treatment. The accurate assessment of liver fibrosis is critical for clinical decision-making and monitoring. Globally, more than 80% of people with cirrhosis develop hepatocellular carcinoma (HCC) [18].

Coco et al. (2007) [19] and Arena et al. (2008) [20] reported that alanine aminotransferase (ALT) and aspartate aminotransferase (AST) levels were elevated in patients with chronic viral hepatitis [21]; however, in the current study, age, serum bilirubin, AST, ALT, ALP, PT, and FibroScan levels were elevated in patients with liver cirrhosis. Our latest study was analogous to Rodrigues (2023) [22] and Sheth et al. (1998) [21], who discovered that the mean serum AST value in cirrhotic patients was significantly greater than in noncirrhotic persons.

According to earlier studies, the changes in total bilirubin count affected LC [23]. In the present study, other factors that affect LC in individuals with CHC were investigated. It was found that patients with CHC-LC have higher age, serum bilirubin, AST, ALT, ALP, PT, and liver stiffness measurement (LSM) value.

In the present study, the correlations between LC and demographic characteristics, as well as LC and the clinical examination of blood, were examined. Some factors (age, BMI, Hb, PLT, bilirubin, ALP, and albumin) showed a negative correlation, while some factors (sex, TLC, and PT) showed a positive correlation, and some other factors (AST and ALT) showed no correlation.

Based on the findings of this study, it is recommended that patients with CHC-LC who have no signs of acute exacerbation or infection with another acute hepatitis be evaluated for overall clinical factors using a medical history, serological examination, and imaging.

Liver stiffness assessment is very useful in the diagnosis of liver fibrosis and cirrhosis, regardless of the source of liver illness. According to the Baveno-7 guidelines, measuring liver stiffness may be an accurate way to estimate the severity of cirrhosis in chronic hepatitis C [24]. In the current study, the FibroScan value of cirrhotic patients ranged from 12.5 to 75.4 kPa, and patients with different stages of liver cirrhosis in the CHC-LC group were found to be as follows: no (0%) patients in stages F0-F1, 17 (17%) patients in stage F2, 29 (29%) patients in stage F3, and 54 (54%) patients in stage F4.

The study investigating risk factors for advanced fibrosis and cirrhosis in CHC patients has some limitations. For example, the sample size may be relatively small, potentially limiting the generalizability of the findings, and there might be a selection bias as the study recruited participants from a specific population, and being retrospective, it could introduce recall bias. Additionally, the study's single-center design might limit its external validity.

## Conclusions

In conclusion, our findings indicate a clear association between advancing age and significant changes in key liver-related parameters. Notably, we observed an upward trend in markers such as serum bilirubin, AST, ALT, ALP, PT, and FibroScan scores, coupled with a decrease in PLT and albumin levels among CHC-infected patients. This heightened surveillance is essential for the early detection of liver-related events, which can be pivotal in improving patient outcomes.

## References

[1] Stasi C, Silvestri C, Voller F (2020). Update on hepatitis C epidemiology: unaware and untreated infected population could be the key to elimination. SN Compr Clin Med.

[2] Patwa AK, Deep A, Kumar S, Rungta S, Atam V, Swaroop S (2021). Previous history of surgery in females and roadside shaving in males are the commonest risk factors for hepatitis C infection: a cross-sectional retrospective study. J Family Med Prim Care.

[3] Farci P, Bukh J, Purcell RH (1997). The quasispecies of hepatitis C virus and the host immune response. Springer Semin Immunopathol.

[4] Perz JF, Armstrong GL, Farrington LA, Hutin YJ, Bell BP (2006). The contributions of hepatitis B virus and hepatitis C virus infections to cirrhosis and primary liver cancer worldwide. J Hepatol.

[5] Mokdad AA, Lopez AD, Shahraz S (2014). Liver cirrhosis mortality in 187 countries between 1980 and 2010: a systematic analysis. BMC Med.

[6] (2024). Global health estimates: leading causes of death. https://www.who.int/data/gho/data/themes/mortality-and-global-health-estimates/ghe-leading-causes-of-death.

[7] de Oliveria Andrade LJ, D'Oliveira A, Melo RC, De Souza EC, Costa Silva CA, Paraná R (2009). Association between hepatitis C and hepatocellular carcinoma. J Glob Infect Dis.

[8] Colapietro F, Maisonneuve P, Lytvyak E (2024). Incidence and predictors of hepatocellular carcinoma in patients with autoimmune hepatitis. J Hepatol.

[9] Vogel A, Meyer T, Sapisochin G, Salem R, Saborowski A (2022). Hepatocellular carcinoma. Lancet.

[10] Malakar S, Pande G, Mishra P, Ghoshal UC (2024). Incidence of hepatocellular carcinoma in patients with autoimmune liver disease in India. J Clin Exp Hepatol.

[11] Prakash S, Jain A, Sankhwar SN (2014). Prevalence of hepatitis B & C viruses among patients on hemodialysis in Lucknow, Uttar Pradesh. Clin Epidemiol Glob Health.

[12] Taneja S, Mehtani R, De A (2023). Spectrum of autoimmune liver disease and real-world treatment experience from a tertiary care hospital. J Clin Exp Hepatol.

[13] Rungta S, Deep AM, Swaroop SU (2019). Malnutrition in liver cirrhosis: a review. J Clin Diag Res.

[14] Rungta S, Kumari S, Deep A, Verma K, Swaroop S (2021). APRI and FIB-4 performance to assess liver fibrosis against predefined Fibroscan values in chronic hepatitis C virus infection. J Family Med Prim Care.

[15] Sandrin L, Fourquet B, Hasquenoph JM (2003). Transient elastography: a new noninvasive method for assessment of hepatic fibrosis. Ultrasound Med Biol.

[16] Saito H, Tada S, Nakamoto N (2004). Efficacy of non-invasive elastometry on staging of hepatic fibrosis. Hepatol Res.

[17] Cheemerla S, Balakrishnan M (2021). Global epidemiology of chronic liver disease. Clin Liver Dis (Hoboken).

[18] Zhang EL, Zhang ZY, Wang SP (2016). Predicting the severity of liver cirrhosis through clinical parameters. J Surg Res.

[19] Coco B, Oliveri F, Maina AM (2007). Transient elastography: a new surrogate marker of liver fibrosis influenced by major changes of transaminases. J Viral Hepat.

[20] Arena U, Vizzutti F, Corti G (2008). Acute viral hepatitis increases liver stiffness values measured by transient elastography. Hepatology.

[21] Sheth SG, Flamm SL, Gordon FD, Chopra S (1998). AST/ALT ratio predicts cirrhosis in patients with chronic hepatitis C virus infection. Am J Gastroenterol.

[22] Rodrigues SG (2023). Baveno VII criteria to predict decompensation in compensated advanced chronic liver disease: still some shades of grey. Clin Mol Hepatol.

[23] Reedy DW, Loo AT, Levine RA (1998). AST/ALT ratio > or = 1 is not diagnostic of cirrhosis in patients with chronic hepatitis C. Dig Dis Sci.

[24] Ohkubo A (1994). [Bilirubin metabolism in liver cirrhosis] (Article in Japanese). Nihon Rinsho.

